# Improving medical leadership education through the Feagin leadership program

**DOI:** 10.5116/ijme.5974.bb0d

**Published:** 2017-08-16

**Authors:** Kevin L. Anderson, Mary In-Ping Huang Cobb, Rathnayaka M. Gunasingha, Nathan H. Waldron, Andrew Atia, James R. Bailey, Joseph P. Doty, Jane H. Boswick-Caffrey, Dean C. Taylor

**Affiliations:** 1 Duke University School of Medicine, Durham, USA; 2 Department of Neurosurgery, Duke University Medical Center, Durham, USA; 3 Department of Anesthesia, Duke University Medical Center, Durham, USA; 4 Department of Orthopedic Surgery, Duke University Medical Center, Durham, USA; 5 Duke Corporate Education, Durham, USA

**Keywords:** Medical leadership education, the Feagin leadership program, leadership program, interdisciplinary teams

## Introduction

With little or no formal training in medical school or residency, physicians today are often unprepared for their leadership roles within large interdisciplinary healthcare teams.^1^^,^^2^  These leadership roles range from leading individual patients to larger interdisciplinary organizations. Physicians are expected to lead effectively based on the assumption that the skills that made them successful as a physician will naturally transfer into success as a leader.^3^ Although this may be true for some physicians, it may not always be the case. Recognizing that many physicians lack the necessary leadership skills, healthcare organizations continue to fill many core leadership positions with skilled non-physician leaders.^4^

The American College of Graduate Medical Education^5^ (ACGME) and the Institute of Medicine^6^ (IOM) have acknowledged the need to develop physician leaders. The IOM has mandated academic medical centers to “develop leaders at all levels”^6^  who can not only “manage the organizational and systems changes necessary to improve health through innovation in health professions education, patient care, and research,”^6^  but can also “improve integration and foster cooperation within and across the academic health center enterprise.”^5^^,^^6^  However, few medical training centers have such a curriculum in place to make this happen. To help address this deficiency the Feagin Leadership Program was developed at Duke University in 2010 in honor of John A. Feagin, Jr., MD. The purpose of this paper is to describe this program and its positive effect on alumni.

### Medical leadership program

The Feagin Leadership Program is a nine-month longitudinal immersion experience for fellows, residents, and third year medical students. Application and selection are based on leadership potential demonstrated in an essay and letters of recommendation. Scholars participate in a curriculum that includes leadership development seminars, workshops, a leadership-focused team project, individual leadership coaching, and active participation at leadership conferences. Seminar topics include defining personal leadership and values, mentoring, emotional intelligence, ethical leadership, difficult conversations, networking, and executive presence. Scholars are exposed to multiple leadership philosophies and styles with the intent that they will integrate the various ideas into their own experience, and develop their own leadership style.

The learning principles for the Scholars include a definition of leadership based on the ability to influence, and a research-based model of competencies necessary for effective, ethical leadership. We define health and healthcare leadership for the Feagin Leadership Program as the ability to influence others for the benefit of patients and patient populations. A research-based health leadership model provides the framework for the program. The model is comprised of a core principle of patient-centeredness, and core competencies of critical thinking, emotional intelligence, integrity, selfless service, and teamwork.

### Evaluation

Since the initiation of the Feagin Leadership Program, we have noticed a positive influence on the Scholars. To evaluate the program’s effect on the Scholars’ leadership development, a Qualtrics survey was distributed to 80 alumni via email. When scholars were asked to contrast their leadership behavior before and after completing the program, they felt that their skills improved in the areas of critical thinking, emotional intelligence, integrity, selfless service, and teamwork. Medical students saw the largest effect in the domains in their ability to motivate others, manage others, and self-awareness. Resident trainees saw the most improvement in their abilities to optimize team dynamics, effectively communicate, motivate others, be self-aware, and self-manage. Lastly, fellows improved their ability to think holistically, effectively communicate, and be resilient.

### Perspective

The primary goal of medical education is to train current and future physicians to be equipped with effective clinical and decision-making skills. Unfortunately, there are limited resources focused on developing trainees in the area of leadership. This gap, unfortunately, makes many physicians ill-equipped to cope with the current model of medical practice.^7^ Previous studies have found leadership gaps in communication, team building, planning, priority setting, and problem solving.^8^ Many of these leadership gaps are integral parts of the Feagin Leadership Program.

In our evaluation, we found that implementation of a structured medical leadership curriculum improved leadership skills in trainees of all levels. This uniquely structured program may serve as a template for other academic institutions in designing curriculum to address this gap in formal medical leadership training. This program has evolved over the past five years, with a curriculum that has adapted not only to the changing demands of medicine, but also based on the evolving field of medical leadership, and alumni feedback. We have also learned that inclusion of a diverse group of medical specialties with different levels of training has enhanced our understanding of leadership across interdisciplinary teams.

## Conclusions

The Feagin Leadership Program has had a consistent positive influence on its alumni. This effect remained true across different training levels, with interval improvement in critical thinking, emotional intelligence, integrity, selfless service, and teamwork.

### Acknowledgements

The authors wish to thank Dr. Kathryn Andolsek, Assistant Dean of Premedical Education, for assistance in the project and her guidance in target journal selection; Donald T. Kirkendall, ELS for his help in preparing the manuscript for submission; and Dr. John A. Feagin, Jr. for his solid core character and leadership that inspired the development of this program.

### Conflict of Interest

DCT is Chairman, JPD is Executive Director and JHBC serves on the advisory board of the Feagin Leadership Program. JRB is a naval service member and the views expressed in this article don’t reflect the official policy or position of the Department of the Navy. KLA, MHC, RMG, NHW, AA, and JRB have no conflicts of interest to report.
